# Characterizing How Families Cope with Pediatric Misophonia: Youth Coping, Parent Responses, and Associations with Symptom Severity

**DOI:** 10.3390/jcm15207986

**Published:** 2026-10-15

**Authors:** Abigail E. Pine, Bruce E. Compas, Joseph F. McGuire

**Affiliations:** 1 Division of Child & Adolescent Psychiatry, Department of Psychiatry and Behavioral Sciences, Johns Hopkins University School of Medicine, Baltimore, MD 21205, USA; apine1@jh.edu; 2 Department of Psychology and Human Development, Vanderbilt University, Nashville, TN 37203, USA; 3 Developmental Behavioral Health, Kennedy Krieger Institute, Baltimore, MD 21205, USA

**Keywords:** misophonia, coping, youth, parenting

## Abstract

**Background/Objectives:** Given that the evidence-based treatment for pediatric misophonia remains in its early stages, families have developed their own strategies to cope with misophonia-related distress. The control-based model of coping distinguishes between voluntary coping and involuntary responses to stressful events and provides a useful framework for understanding responses to misophonia-related distress. This report sought to characterize the range of coping strategies and involuntary stress responses among youth and families and their associations with misophonia symptom severity to inform novel targets for future family-focused interventions. **Methods:** Participants were recruited online across the United States and included 85 parents (*M* = 42.58 years, *SD* = 6.25) who reported on their children (*M* = 12.92 years, *SD* = 2.34) with misophonia symptoms. Parents completed questionnaires about their child’s misophonia symptoms, coping and involuntary stress responses, parent responses to symptoms, perceived effectiveness of approaches, parenting stress, and family functioning. **Results:** Across misophonia measures, greater primary and secondary control coping were associated with lower misophonia symptom severity (*rs* = −0.31 to −0.59, *ps* ≤ 0.004), whereas involuntary engagement and involuntary disengagement were associated with greater symptom severity (*rs* = 0.29 to 0.60, *ps* ≤ 0.007). Associations between disengagement coping and symptom severity were less consistent across measures. More frequent parent responses to misophonia were associated with greater misophonia symptom severity across measures (*rs* = 0.45 to 0.65, *ps* < 0.001). **Conclusions:** A range of strategies are utilized by youth and families to manage misophonia-related distress, which have unique connections to misophonia symptom severity. Family-focused interventions may benefit from promoting flexible, collaborative strategy use aimed at reducing impairment, rather than categorizing strategies as uniformly “adaptive” or “maladaptive.”

## 1. Introduction

Misophonia is a condition characterized by intense emotional, physiological, and/or behavioral responses to specific sounds or their associated stimuli [1,2]. Symptoms typically onset during childhood [1] and can profoundly impact both affected youth and their families [3,4,5,6,7]. Existing evidence-based treatments for youth with misophonia remain limited, with initial findings suggesting promise for cognitive-behavioral (CBT) interventions [8,9,10,11]. However, in the absence of a definitive evidence-based treatment, many families have needed to develop their own strategies to manage misophonia-related distress. As such, it is critical to understand and characterize the range of strategies used by youth and families, as it can identify promising targets for future family-focused interventions.

Existing CBT models highlight the role of intrusive or automatic thoughts, avoidance, and physiological responses to triggering sounds, which may be addressed through a range of strategies, including graded exposure, cognitive restructuring, distress-tolerance skills, and mindfulness techniques [12,13]. However, evidence from randomized controlled trials remains limited [10], and most, but not all [9,14], trials examining treatments for misophonia have been conducted with adults [13,15,16,17]. Importantly, prior work cautions against uniformly applying CBT principles without considering features that may be specific to misophonia, as well as individual differences in symptom presentation and function [12,18]. Such considerations may be particularly important when applying CBT principles to youth, given developmental differences in emotional and cognitive capacities and the broader family contexts in which symptoms occur.

Although research on pediatric misophonia is still in its early stages, decades of research on stress and coping in children and adolescents [19,20] can provide a valuable framework for understanding how youth respond to misophonia-related distress. This work distinguishes between voluntary coping and involuntary responses to stressful events. Coping refers to “conscious and volitional efforts to regulate emotion, cognition, behavior, physiology, and the environment in response to stressful events or circumstances” [19] (p. 89), whereas involuntary responses reflect reactions to stress that are less directly under conscious control, including those influenced by temperament or conditioned responses. The control-based model of coping [21] further distinguishes three categories of coping: (1) *primary control coping*, which involves efforts to directly alter the stressor or one’s emotional response to it; (2) *secondary control coping*, which involves efforts to adapt to aspects of the problem that cannot be directly changed; and (3) *disengagement coping*, which involves efforts to orient away from the stressor. This model also identifies two categories of involuntary stress responses: (1) *involuntary engagement*, including intrusive thoughts, rumination, and emotional or physiological arousal; and (2) *involuntary disengagement*, including emotional numbing, cognitive interference, and escape behaviors. Together, both coping and involuntary stress responses capture a wide range of the ways that youth respond to stress. This model has been applied across diverse populations experiencing chronic stress, including youth living in poverty, serious medical conditions (e.g., cancer), and mental and behavioral health conditions [22,23,24,25].

**Figure 1:**
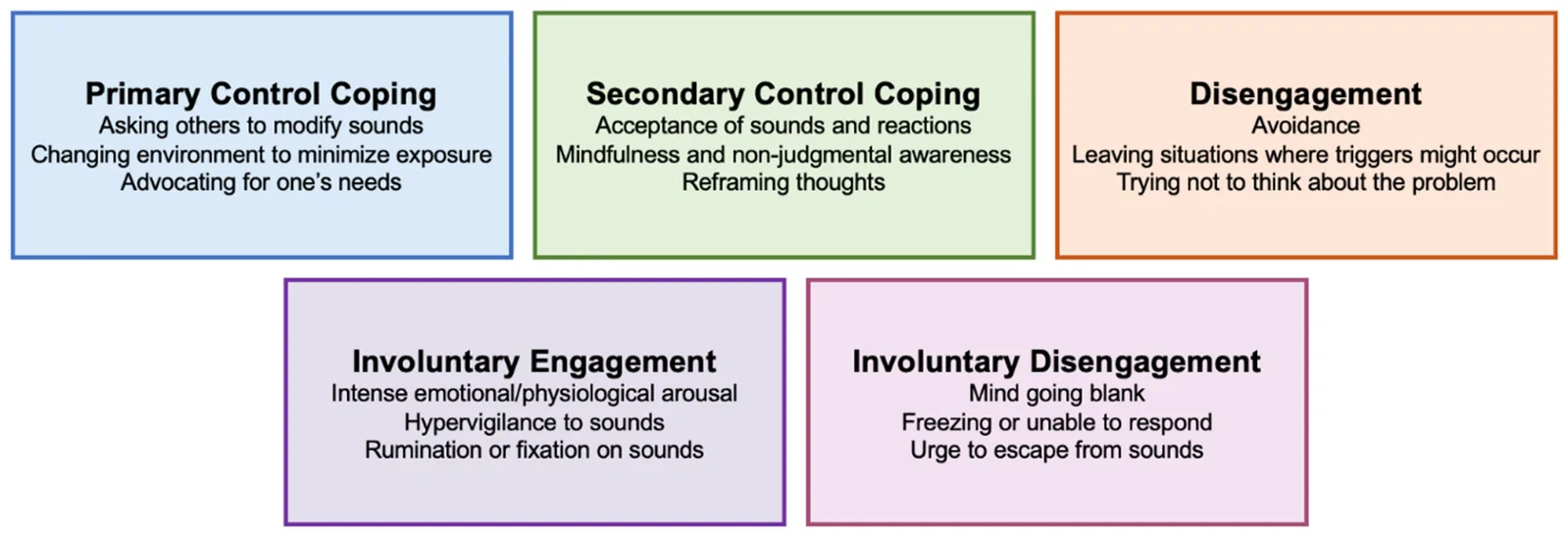
The control-based model of coping applied to youth with misophonia.

The control-based model of coping is particularly well-suited to understanding misophonia because both distressing sounds and reactions are often unpredictable and difficult to control in everyday environments. Figure 1 illustrates how these youth responses may present in the context of pediatric misophonia, as reactions to misophonia triggers can involve both involuntary stress responses (e.g., physiological arousal, hypervigilance) and voluntary coping efforts (i.e., using headphones, avoiding distressing situations). Prior research using the control-based model has found significant associations between primary and secondary control coping and lower psychopathology symptom severity, as well as between disengagement coping and higher levels of symptoms [20]. However, no prior study has evaluated the applicability of this model for misophonia or examined the role of parent responses in supporting youths’ coping and stress responses in this population. Integrating this coping model with an understanding of how parent responses relate to youths’ coping and stress responses can identify potential targets for interventions designed to reduce functional impairment and promote resilience over time.

Family processes are central to understanding how youth cope with different sources of stress, and parents play a crucial role in the development of youth coping skills [26,27]. Parent responses—such as reinforcing certain strategies, modeling specific responses, and providing emotional validation—can have a powerful influence on how youth learn to manage stress. For example, consider a 9-year-old child who experiences intense distress in response to hearing his younger sister chew during dinner. His parents may support primary control coping by changing his seat so that he sits farther from his sister, facilitate secondary control coping by playing his favorite music as a distraction, or support disengagement coping by coordinating separate mealtimes for the siblings. Beyond parent responses, features of the broader family context, including family functioning and parenting stress, can also have an important impact on how parents respond to youth distress [28,29,30]. Research that integrates the lived experiences of parents can provide valuable insights into the perceived effectiveness of specific responses, as well as how such responses may be connected to youth responses, symptom severity, and broader family context.

**Figure 2:**
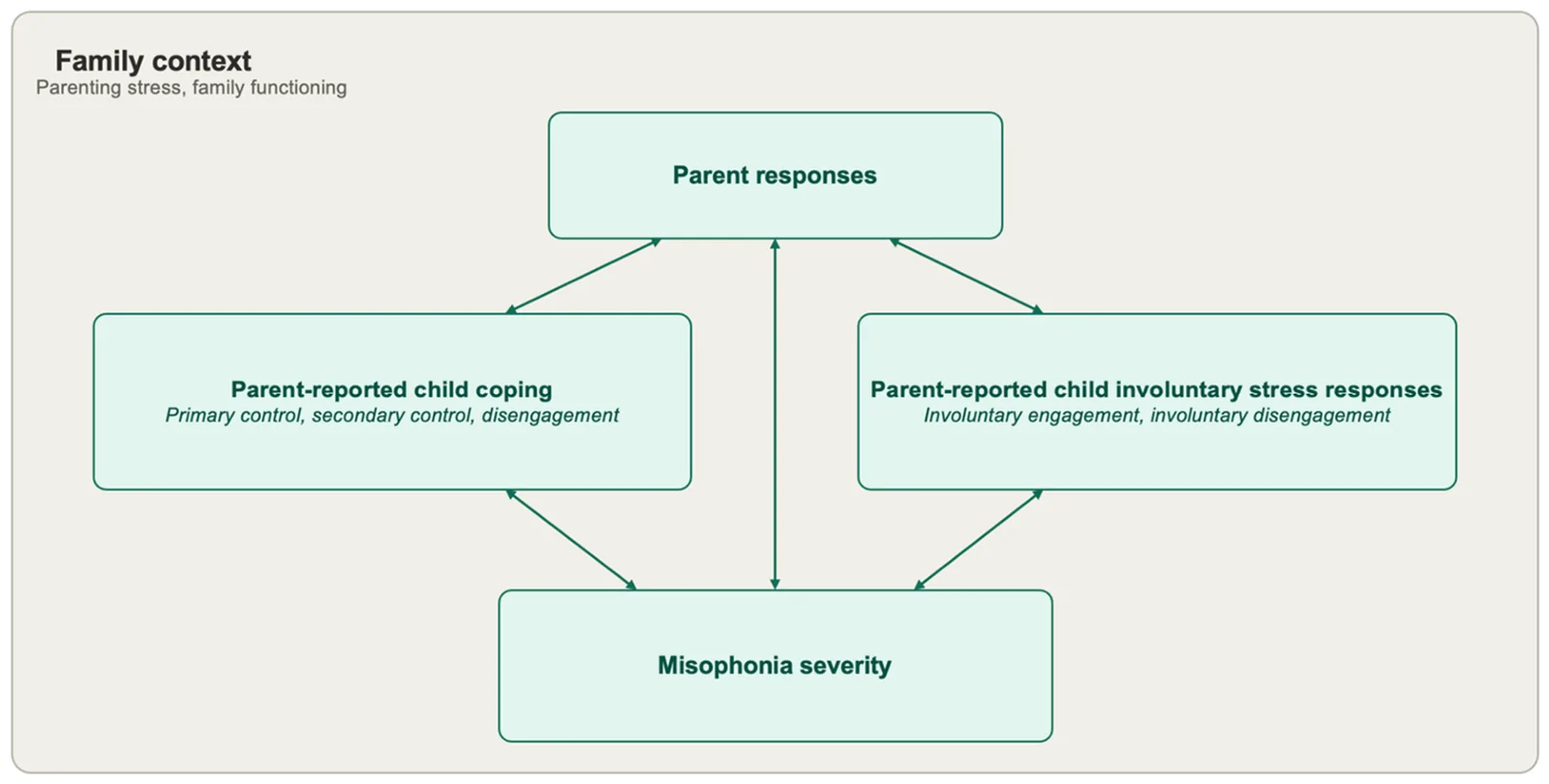
Conceptual framework of associations among youth responses, parent responses, family context, and misophonia symptom severity. Note. Bidirectional arrows represent hypothesized associations among constructs and do not imply causal relationships.

The current study addresses existing gaps by applying the control-based coping model to characterize how youth and families respond to misophonia-related distress. First, this study describes youth responses, parent responses, and parents’ perceived effectiveness of these approaches to manage misophonia present among youth. Second, we examine associations among youth responses, parent responses, and misophonia symptom severity. Finally, we explore how broader family context variables—including parenting stress and family functioning—relate to identified youth and parent processes. Based on prior work applying the control-based coping model, we hypothesized that greater primary and secondary control coping would be associated with lower symptom severity, whereas greater disengagement coping, involuntary engagement, and involuntary disengagement would be associated with higher symptom severity. Figure 2 presents a framework of hypothesized associations among these constructs and is not intended to imply temporal or causal relationships.

## 2. Method

### 2.1. Participants and Procedures

Parents of youth with misophonia symptoms (*N* = 85) were recruited online through misophonia organizations (e.g., Misophonia Research Fund), social media (e.g., Parents of Children with Misophonia Facebook Groups), and relevant listservs (e.g., SoQuiet). Families were eligible if they resided in the United States, and a parent reported having a child aged 8–17 with misophonia symptoms on a parent-reported version of the Duke-Vanderbilt Misophonia Screening Questionnaire [31], administered as part of the study screening procedures. If eligible, parents provided informed consent under a waiver of documentation of consent and subsequently completed a battery of survey measures via REDCap. Parents were compensated $40 for completing the survey battery. All study procedures were approved by the Johns Hopkins University School of Medicine Institutional Review Board (IRB00523773).

### 2.2. Measures

The Amsterdam Misophonia Scale (A-MISO-S) [32] is a six-item measure modeled after the gold-standard measure of obsessive-compulsive symptom severity, the Yale-Brown Obsessive-Compulsive Scale (Y-BOCS) [33]. The A-MISO-S assesses several domains, including time occupied by symptoms, control over thoughts, distress, and impairment, with items rated from 0 (*none*) to 4 (*extreme*). The A-MISO-S was adapted for the parent report in the current study by replacing “you/your” with “your child” throughout. Although the A-MISO-S has demonstrated good internal consistency and construct validity in adults [34], work with youth samples indicates that item four—assessing efforts to resist misophonia thoughts—does not consistently load onto the overall factor structure [3,35]. Accordingly, a total score comprising the five remaining items was used and demonstrated good internal consistency in the current sample (Cronbach’s α = 0.85).

The Misophonia Assessment Questionnaire (MAQ) [36] is a 21-item measure assessing the negative impact of misophonia on emotions, thoughts, and activities. Items are rated from 0 (*not at all*) to 3 (*almost all the time*). Consistent with prior work [3,35], the MAQ was adapted for parent reports by asking parents to replace “my sound issues” with “my child’s sound issues” when completing the measure. Prior work has demonstrated good convergent validity and internal consistency [35]. Internal consistency in the current sample was α = 0.94.

The Duke-Vanderbilt Misophonia Screening Questionnaire (DVMSQ) [31] is an 18-item questionnaire assessing the symptoms of misophonia proposed in the Revised Amsterdam Criteria [37] and associated functional impairment. The measure contains an initial screening item, “*Are there specific sounds that your child is extremely bothered by, even if they are not loud?*” If answered affirmatively, parents completed additional items about misophonia symptoms and interference, rated from 0 (*never; not at all*) to 4 (*very often; an extreme amount*). While the DVMSQ has been previously validated in adults [31,38], it was adapted for parent report in the current study by replacing “you/your” with “your child.” Internal consistency in the current sample was strong (α = 0.95).

The Duke Misophonia Questionnaire (DMQ) [39] is an 86-item measure comprising 9 subscales, including affective responses, physiological responses, and cognitive responses to misophonia. The sum of these three subscales (23 items) was used as a symptom severity composite in the current study. Items are rated from 0 (*never*) to 4 (*always/almost always*). The DMQ has been previously validated for use in adults [39,40], and it was adapted for parent report in the current study by replacing “you/your” with “your child.” Because internal cognitions are challenging for parents to observe, cognitive items were further modified to ask, “*In the past month on average, when intensely bothered by a sound or sounds, please rate how often your child expressed each of the following thoughts*.” The internal consistency of the 23-item symptom severity composite in the current sample was α = 0.91. The four parent-report misophonia measures (A-MISO-S, MAQ, DVMSQ, and DMQ) were strongly and positively correlated (*rs* = 0.64–0.81, all *ps* < 0.001), providing preliminary evidence of convergent validity among adapted measures.

The Responses to Stress Questionnaire (RSQ) [21] assessed parent-reported youth coping and involuntary stress responses to misophonia-related distress. Parents first rated stressors their child had experienced related to misophonia in the past six months, including “*avoiding meals with family or others because of sounds*,” “*feeling misunderstood or not believed about misophonia*,” and “*feeling embarrassed or ashamed about reactions to sounds*.” The second and primary part of the RSQ contains 57 items assessing how youth responded to these stressors over the past six months, rated from 1 (*not at all*) to 4 (*a lot*). Consistent with the overall structure of the RSQ, items were tailored to the specific domain of stress examined (i.e., misophonia) while retaining the same underlying structure as prior versions of the measure. The RSQ generates five factors: *primary control coping* (problem solving, emotion regulation, and emotional expression); *secondary control coping* (positive thinking, acceptance, cognitive reappraisal, and distraction); *disengagement coping* (denial, avoidance, and wishful thinking); *involuntary engagement* (rumination, intrusion, emotional arousal, physical arousal, and impulsivity); and *involuntary disengagement* (emotional numbing, cognitive interference, fleeing, and inaction).

Factors are calculated as proportion scores to account for individual differences in overall rates of coping and stress response items. Additionally, for the current study, parents rated the perceived effectiveness of broad coping categories (e.g., problem solving, emotion regulation, emotional expression, thinking strategies, distraction, avoidance, advocacy/communication, and rumination) in helping their child cope with misophonia, from 0 (*not at all helpful*) to 4 (*extremely helpful*). The factor structure, reliability, and validity of the RSQ have been supported across diverse samples [22,24,41]. Internal consistency for the five factors in the current sample ranged from α = 0.69 for primary control to α = 0.94 for secondary control coping. The full misophonia-adapted RSQ can be found in the Supplemental Materials (Supplementary File S1).

The Family Accommodation Scale for Misophonia (FAS) is a 13-item parent-report measure assessing parent responses to misophonia symptoms, including participation in symptoms, assisting with avoidance, and providing items needed in response to symptoms (e.g., headphones). The FAS was originally developed to assess family accommodation in obsessive-compulsive disorder (OCD) and anxiety [42,43], and has recently been used to characterize parent responses to youth misophonia [44]. A sum score of the first nine items, which assesses frequency of parent responses, was used. Parents also rated the perceived effectiveness of each type of response in helping their child cope with misophonia, from 0 (*not at all helpful*) to 4 (*extremely helpful*). In contrast to prior work [44] (α = 0.67), internal consistency for the nine-item frequency score was good (α = 0.81).

The Parenting Stress Scale (PSS) [45] is an 18-item parent-report measure of parenting stress capturing domains of rewards, stressors, lack of control, and satisfaction. Items are rated on a scale from 1 (*strongly disagree*) to 5 (*strongly agree*). The PSS has demonstrated strong reliability and validity for examining parenting stress in diverse populations [46], and internal consistency in the current sample was α = 0.90.

The Family Assessment Device—General Functioning Subscale (FAD-GF) [47] is a 12-item subscale of the McMaster Family Assessment Device designed to assess overall “health/pathology” of a family. Items are scored on a scale from 1 (*strongly agree*) to 4 (*strongly disagree*). The FAD-GF has demonstrated good psychometric properties when used as a standalone measure [48,49]. Internal consistency in the current sample was α = 0.90.

### 2.3. Data Analytic Plan

**Figure 3:**
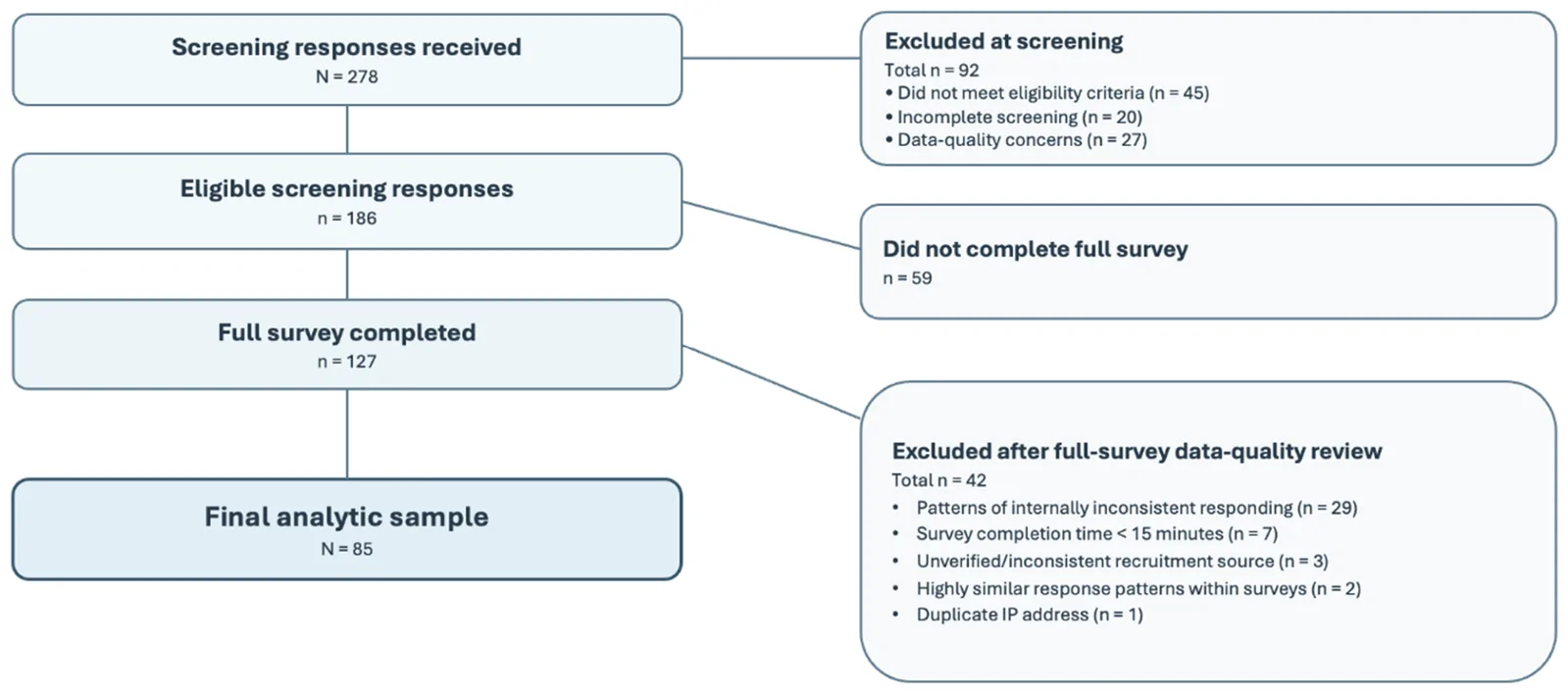
Participant flow and data quality screening. Note. Data quality concerns at the screening level included duplicate email or IP addresses, as well as highly similar response patterns across multiple screening submissions completed within a short time frame.

Analyses were conducted in SPSS, version 31 [50]. Prior to data analysis, completed survey responses were examined for data quality. Responses were excluded based on indicators of potentially invalid responses, including patterns of internally inconsistent responses across conceptually overlapping items, survey completion in less than 15 min, and highly similar response patterns within surveys. A total of 42 completed responses were excluded based on these criteria (see Figure 3). Bivariate correlations were used to examine associations among youth responses, parent responses, misophonia symptom severity, parenting stress, and family functioning. Three linear regression models examined associations between youth and parent responses and misophonia symptom severity. The coping model included primary control, secondary control, and disengagement coping as simultaneous predictors of symptom severity. The involuntary stress response model included involuntary engagement and involuntary disengagement as simultaneous predictors. The parent response model included the nine-item FAS frequency score as the predictor.

To explore whether associations between youth responses and symptom severity varied as a function of parent responses, two hierarchical regression models were conducted. Main effects were entered in the first step, followed by interaction terms between parent responses and either (1) coping domains (primary control, secondary control, and disengagement) or (2) involuntary stress response domains (involuntary engagement and involuntary disengagement). Predictors were mean centered prior to generating interaction terms, and multicollinearity was evaluated using variance inflation factors (VIFs) and tolerance statistics. Given the final analytic sample (*N* = 85), sensitivity analyses indicated 80% power to detect incremental effects of *f*^2^ = 0.12–0.14 across moderation models. Significant interactions were probed by examining simple slopes at low (−1 *SD*), mean, and high (+1 *SD*) levels of parent responses [51]. All regression analyses included A-MISO-S—selected a priori as the primary measure of misophonia symptom severity. The Benjamini-Hochberg false discovery rate procedure (*q* = 0.05) was applied separately to two families of correlation comparisons: (1) associations between youth and parent responses and (2) associations of family context (parenting stress and family functioning) with youth and parent responses. Regression analyses were considered significant at *p* < 0.05.

## 3. Results

### 3.1. Sample Characteristics

Participants included 85 parents (*M* = 42.58 years, *SD* = 6.25), reporting on their children (*M* = 12.92 years, *SD* = 2.34). Surveys were completed by 60 mothers and 24 fathers, with parent type missing for one participant. Youth were 46 (54.1%) female and 39 (45.9%) male. Among youth, 71 (83.5%) were White, 8 (9.4%) were Black or African American, 1 (1.2%) was Native Hawaiian/Pacific Islander, and 5 (5.9%) were multiracial. Four youth (4.7%) identified as Hispanic or Latinx. Among parents with available race data (*n* = 84), 72 (85.7%) were White, 8 (9.5%) were Black or African American, 1 (1.2%) was Native Hawaiian/Pacific Islander, and 3 (3.6%) were multiracial. Two parents (2.4%) were Hispanic or Latinx. Responses were received from 29 U.S. states. On average, parents reported moderate symptoms of misophonia present among youth (*M* = 11.25, *SD* = 3.44; *range*: 5–20) on the A-MISO-S. Table 1 presents additional details on sample characteristics.

### 3.2. Characterizing Youth and Parent Responses

Full descriptive statistics for youth coping and stress responses, parent responses, and perceived effectiveness are presented in Table 2. Across RSQ domains, involuntary engagement accounted for the largest proportion of youth stress responses, followed by involuntary disengagement, secondary control coping, primary control coping, and disengagement coping. Avoidance and problem-solving/environmental modification were perceived by parents as the most helpful strategies for managing youths’ misophonia-related distress, whereas rumination/getting stuck on misophonia-related thoughts was perceived as the least helpful.

Among parent responses, modifying one’s own behavior (e.g., speaking more quietly or minimizing sounds while eating or breathing), assisting in avoidance of potential misophonia triggers, providing reassurance, and modifying family routines were reported most frequently. Modifying one’s own behavior, assisting in avoiding potential triggers, modifying family routines, and providing items needed such as headphones were perceived as the most helpful parent responses.

### 3.3. Associations Among Youth Responses, Parent Responses, and Misophonia Severity

Descriptive statistics and correlations among study variables are presented in Table 3. Across measures of misophonia severity, greater primary and secondary control coping were consistently associated with lower misophonia symptom severity (*rs* = −0.31 to −0.59, *ps* ≤ 0.004), whereas involuntary engagement and involuntary disengagement were consistently associated with greater symptom severity (*rs* = 0.29 to 0.60, *ps* ≤ 0.007). Associations between disengagement coping and symptom severity were less consistent across measures. Specifically, greater disengagement coping was associated with lower symptom severity on the A-MISO-S (*r* = −0.21, *p* = 0.049) but was not significantly associated with other measures of misophonia symptom severity. More frequent parent responses on the FAS (e.g., providing reassurance, assisting with avoidance) were associated with greater misophonia symptom severity across measures (*rs* = 0.45 to 0.65, *ps* < 0.001). When examining associations between youth and parent responses, no correlations remained significant after correcting for multiple comparisons.

Associations between youth and parent responses and symptom severity were further examined using linear regression models. Across all models, collinearity diagnostics indicated no evidence of concerning multicollinearity (VIFs = 1.11–1.42; tolerances = 0.70–0.90). Regression diagnostics also indicated no significant violations of normality or heteroscedasticity. In the coping model, the overall model was significant, *F* = 20.50, *p* < 0.001, accounting for 43.2% of the variance in A-MISO-S scores. Greater primary control coping (β = −0.40, *p* < 0.001), secondary control coping (β = −0.44, *p* < 0.001), and disengagement coping (β = −0.24, *p* = 0.01) were each independently associated with lower misophonia symptom severity. In the involuntary stress response model, the overall model was also significant, *F* = 27.94, *p* < 0.001, accounting for 40.5% of the variance in A-MISO-S scores. Greater involuntary engagement (β = 0.35, *p* < 0.001) and involuntary disengagement (β = 0.38, *p* < 0.001) were independently associated with greater misophonia symptom severity. Finally, more frequent parent responses to symptoms were associated with greater misophonia symptom severity (β = 0.55, *p* < 0.001), accounting for 30.2% of the variance in symptom severity, *F* = 35.98, *p* < 0.001.

**Figure 4:**
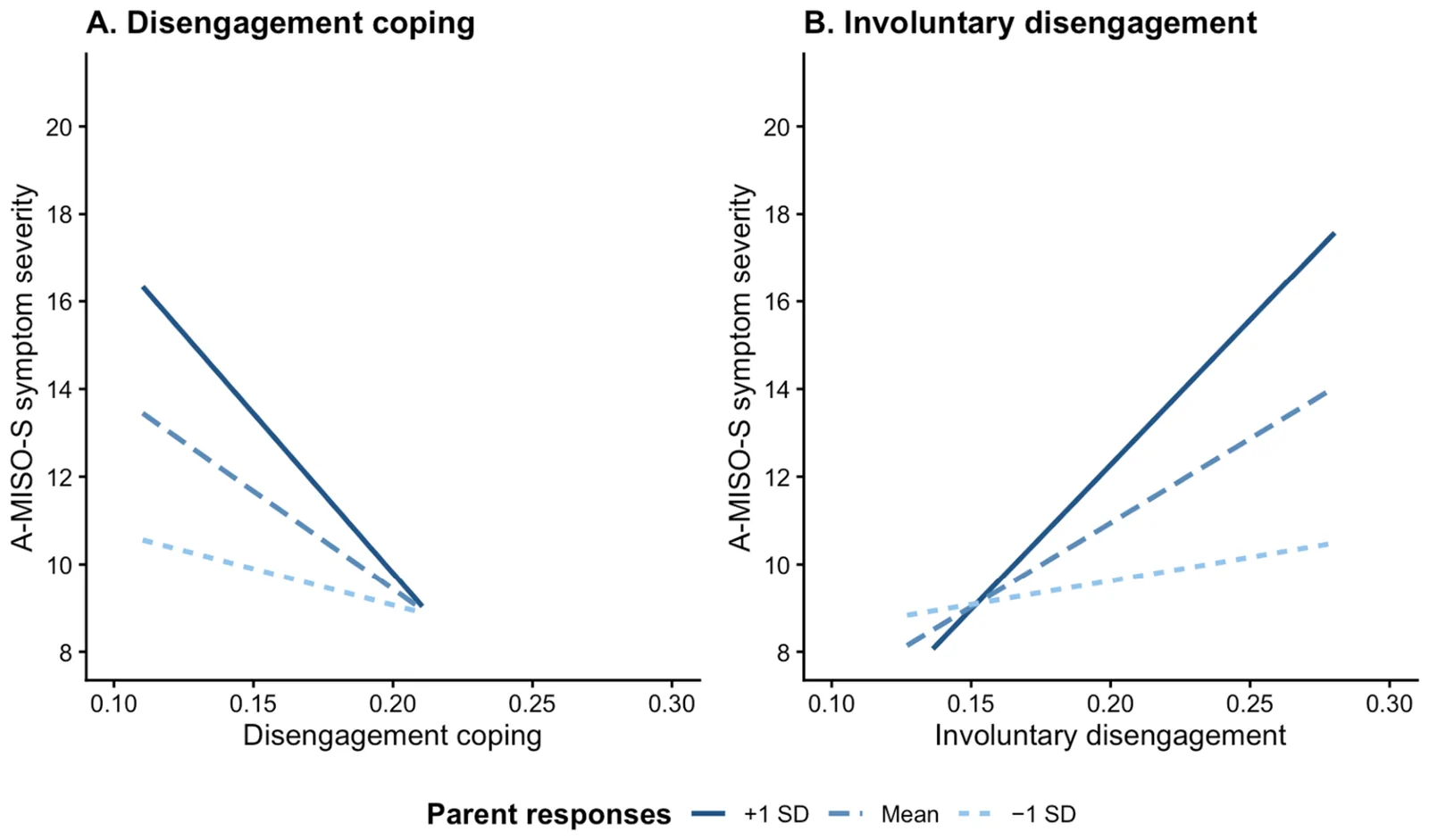
Parent responses moderate associations between youth responses and misophonia symptom severity. Note. Disengagement coping (**A**) and involuntary disengagement (**B**) scores are reported as proportion scores, reflecting the proportion of total responses accounted for in each category.

Hierarchical regression analyses further examined whether associations between youth responses and misophonia symptom severity varied as a function of parent responses. In the coping model, adding the coping × parent response interaction terms did not significantly improve model fit, Δ*R*^2^ = 0.03, Δ*F* = 1.85, *p* = 0.15, relative to the main effect model. However, the disengagement coping × parent response interaction was significant (β = −0.18, *p* = 0.03), whereas interactions involving primary control (β = −0.13, *p* = 0.18) and secondary control coping (β = 0.02, *p* = 0.77) were not. Simple slopes analyses indicated that greater disengagement coping was associated with lower symptom severity at mean and high (+1 *SD*) levels of parent responses, but not at low (−1 *SD*) levels of parent responses (Figure 4A). In the involuntary stress response model, adding the involuntary stress responses × parent response interaction term significantly improved model fit, Δ*R*^2^ = 0.04, Δ*F* = 4.07, *p* = 0.02. The involuntary disengagement × parent response interaction was significant (β = 0.23, *p* = 0.01), whereas the involuntary engagement × parent response interaction was not (β = −0.10, *p* = 0.22). Simple slopes analyses showed that greater involuntary disengagement was associated with greater symptom severity at mean and high (+1 *SD*) levels of parent responses, but not at low (−1 *SD*) levels of parent responses (Figure 4B).

### 3.4. Associations Among Youth Responses, Parent Responses, and Family Context

Among family context variables, greater parenting stress was associated with lower parent-reported youth primary control coping (*r* = −0.36, *p* < 0.001), lower secondary control coping (*r* = −0.37, *p* < 0.001), and both greater involuntary engagement (*r* = 0.42, *p* < 0.001) and involuntary disengagement (*r* = 0.42, *p* < 0.001). Parenting stress was not significantly associated with disengagement coping (*r* = −0.11, *p* = 0.31) or parent responses (*r* = 0.17, *p* = 0.11). After correction for multiple comparisons, there were no significant associations between family functioning and youth or parent responses.

## 4. Discussion

This study aimed to characterize how youth respond to misophonia-related distress using a well-established control-based coping model. In addition to examining youth coping and involuntary stress responses through this framework, the present study extends this work by considering parent responses alongside youths’ responses to misophonia-related distress. Four main findings emerged: (1) Families reported a wide range of voluntary coping strategies, involuntary stress responses, and parenting responses to manage misophonia-related distress; (2) greater primary and secondary control coping were associated with lower symptom severity, whereas greater involuntary stress responses were associated with higher symptom severity; (3) disengagement coping was not consistently associated with symptom severity across measures; and (4) parent responses were both directly associated and interacted with youth responses in relation to symptom severity. With limited existing evidence-based interventions for pediatric misophonia, findings suggest that both youth coping and parenting responses may serve as hypothesized targets for family-focused treatments. Additional considerations for interpreting findings and guiding subsequent intervention development are described.

First, findings suggest that responses to misophonia-related distress are characterized by both voluntary coping and involuntary stress responses. Involuntary engagement (e.g., physiological arousal, hypervigilance to sounds) and involuntary disengagement (e.g., urges to escape from sounds, mind going blank) were the most prevalent endorsed responses. These responses are consistent with the automatic, physiological arousal that frequently characterizes reactions to triggering sounds [52,53,54]. This contrasts with secondary control (e.g., distraction, acceptance of sounds and reactions), primary control (e.g., environmental modification, problem solving), and disengagement (e.g., avoidance, trying not to think about misophonia) coping, which involve deliberate efforts to regulate emotion, thoughts, behavior, physiology, and the environment in response to misophonia-related stressors. Interventions that aim to target responses to sounds should consider which aspects of responses youth can reasonably learn to regulate.

Second, given the consistent associations between greater primary and secondary control coping and lower symptom severity across misophonia measures, interventions that increase primary and secondary control coping may represent one potential pathway for reducing misophonia severity. Indeed, interventions targeting primary and secondary control coping in other child and adolescent populations have demonstrated efficacy in preventing and treating psychopathology [55,56,57,58,59]. Importantly, however, prior work in this area suggests that the effectiveness of individual coping strategies may vary depending on the controllability of the stressor. Specifically, primary control coping, such as changing seats at the dinner table or asking others to stop making a sound, may be especially useful for aspects of a triggering situation that can be changed, whereas secondary control coping, including distraction through listening to music and acceptance of sounds and reactions, may be more helpful for aspects that cannot be changed. Overall, in addition to understanding youths’ capacity to regulate their own responses, the controllability of misophonia stressors has important implications for the development of interventions aimed at optimizing coping.

Third, results demonstrate mixed findings regarding the extent to which disengagement coping is associated with symptom severity. Prior work in adults with misophonia similarly suggests a complex role of disengagement, with greater avoidance associated with better functioning in some domains of quality of life [60]. Notably, this pattern differs from prior meta-analytic work [20] and findings in pediatric anxiety and OCD, whereby greater avoidance has been consistently linked to increased symptoms and functional impairment [61,62]. Although anxiety, OCD, and misophonia share several features, including hypervigilance, avoidance behaviors, and physiological arousal, mounting evidence suggests that misophonia is a distinct condition [63,64] requiring unique adaptations to existing cognitive-behavioral interventions [18]. The high prevalence of avoidance in pediatric misophonia [3,4], coupled with the low acceptability of exposure as an intervention among patients [18,65], further highlights the importance of interventions that promote a flexible and collaborative approach to coping, rather than categorizing avoidance strategies as uniformly adaptive or maladaptive.

Fourth, findings highlight the critical role of parent responses in understanding misophonia symptom severity, as more frequent parent responses were associated with greater symptom severity. Interestingly, in addition to being directly associated with misophonia symptom severity, parent responses moderated associations between both forms of disengagement and misophonia severity. At mean and high, but not low, levels of parent responding, greater involuntary disengagement was associated with higher symptom severity, whereas greater disengagement coping was associated with lower symptom severity. These response patterns can be further understood within a broader family context, such that greater parenting stress, but not family functioning, was linked to lower primary and secondary control coping and greater involuntary engagement and disengagement stress responses. At the same time, the cross-sectional design limits conclusions about the direction of these effects; while it is possible that higher levels of parent responding contribute to greater misophonia severity, it is equally plausible that youth with more severe misophonia symptoms may elicit more frequent responding from parents. In particular, youth with more severe misophonia symptoms may require additional parental support, including greater modification of family routines, assistance avoiding distressing sounds, and availability of items such as headphones to manage symptoms. Although longitudinal research is needed to elucidate temporal relationships among youth responses, family context, and misophonia severity, preliminary evidence suggests that parental involvement warrants consideration in interventions designed to support youth coping.

Taken together, these findings, considered alongside prior work, inform several potential hypotheses for future intervention development: (1) responses to sound triggers involve both involuntary stress responses and voluntary coping responses, with coping being potentially more amenable to modification; (2) coping strategy selection may be most beneficial when matched to the controllability of a particular stressor; (3) parent involvement may support proactive, rather than reactive, coping; and (4) coping strategies should be selected in alignment with family goals. Consider again the 9-year-old child experiencing distress in response to his sister’s eating sounds. If eating together is an important goal for this family, a proactive coping plan could be implemented during dinner. This may include changes to aspects of the situation that are controllable, including parents having ear plugs readily available, as well as planned strategies for aspects that cannot be changed, such as intentionally shifting attention toward family conversation or the taste of food when sounds are noticed. Coping efforts are supported by parents’ praise—directed specifically at increasing the use of identified coping strategies rather than reducing involuntary responses to triggers. Given the cross-sectional nature of the current findings, additional research is needed to determine whether this type of intervention may help youth remain engaged in select meaningful activities by increasing independent coping and supporting parents in scaffolding these approaches.

## 5. Limitations and Future Directions

As this study is the first to apply an established control-based coping framework to pediatric misophonia, several limitations should be considered when interpreting findings. Most importantly, all analyses were cross-sectional and preclude conclusions about causality or directionality. While coping may influence misophonia severity, symptom severity may also shape how youth cope. Relatedly, findings are limited in that they only examine associations with symptom severity and do not consider other important indicators of functioning or quality of life over time. When considering study methodology, not all parent-report symptom measures were validated, internal consistency for primary control coping was relatively low (α = 0.69), and all data were collected online. Although efforts were taken to establish preliminary convergent validity and minimize biased and inaccurate responses, respectively, remote survey administration limited control over the conditions in which participants were completing measures. Moreover, parents were the sole informants on all measures and may not have comprehensive insight into their child’s internal coping processes. Shared method variance, as well as some conceptual overlap between items assessing misophonia symptoms and coping (e.g., avoidance), may have inflated significant associations among parent-reported constructs. In addition, the relatively small sample size may have limited our ability to reliably detect smaller interaction effects, so non-significant interactions should be interpreted with caution.

Parents were recruited from misophonia-affiliated sources, and therefore findings may overrepresent families who have identified their child’s difficulties as misophonia, are actively seeking support or engaged with relevant resources, have children with more severe symptoms, or demonstrate greater parental engagement. As such, the strength of observed associations, especially between parent responses and misophonia symptom severity, may not generalize to broader samples of parents of youth with misophonia. Finally, given that this is the first application of the control-based coping model to misophonia, some strategies central to coping with misophonia (e.g., headphone use) do not cleanly map onto specific coping or involuntary stress response categories. The function and context of such strategies may be as important as the behavior itself. For example, headphone use may allow a child to remain engaged in a valued or necessary activity despite the presence of trigger sounds, whereas in another context it may facilitate avoidance or disengagement from activities. Future research may benefit from differentiating specific coping behaviors from their function to determine whether the control-based coping framework adequately captures coping processes unique to misophonia.

These limitations provide several directions for future research. Most notably, additional multi-informant, longitudinal studies aimed at replicating and extending findings in larger samples are necessary to understand the impact of coping and involuntary stress responses on a range of clinically meaningful outcomes over time (e.g., symptom severity, engagement in daily activities, quality of life). The cross-sectional associations between coping and involuntary stress responses and symptom severity may differ from how these approaches relate to symptoms and other relevant outcomes over time. Future research may also benefit from measuring real-time coping in the context in which triggering sounds occur. Recent work in adults with misophonia has highlighted the importance of ecological momentary assessment (EMA) measures to capture emotion regulation processes in the context of individuals’ daily lives [66,67]. Lastly, although findings from the current study can inform future intervention development, piloting new interventions, alongside iterative feedback from families, is critical to evaluate feasibility and acceptability while minimizing unintended harm [18,68].

## 6. Conclusions

Ultimately, results indicated that greater primary and secondary control coping were associated with lower misophonia symptom severity, whereas greater involuntary engagement and involuntary disengagement were associated with greater symptom severity. More frequent parent responses to misophonia were also associated with greater misophonia symptom severity across measures. Given the cross-sectional design, prospective studies are needed to clarify the longitudinal relationships among involuntary stress responses, coping, and parent responses, as well as their implications for intervention. Findings provide initial support for applying the control-based model of coping to misophonia and offer a framework for informing the development and testing of family-focused interventions aimed at supporting resilience in youth with misophonia and their families.

## Figures and Tables

**Table 1:** Sample characteristics.

	*n* (%)
Parent characteristics	
Age, years; *M* ± *SD*	42.58 ± 6.25
Sex	
Female	60 (71.4%)
Male	24 (28.6%)
Race/Ethnicity	
White	72 (85.7%)
Black or African American	8 (9.5%)
Native Hawaiian/Pacific Islander	1 (1.2%)
Multiracial	3 (3.6%)
Hispanic or Latinx	2 (2.4%)
Youth characteristics	
Age, years; *M* ± *SD*	12.92 ± 2.34
Sex	
Female	46 (54.1%)
Male	39 (45.9%)
Race/Ethnicity	
White	71 (83.5%)
Black or African American	8 (9.4%)
Native Hawaiian/Pacific Islander	1 (1.2%)
Multiracial	5 (5.9%)
Hispanic or Latinx	4 (4.7%)

**Table 2:** Youth Coping, Parent Responses, and Perceived Effectiveness of Approaches.

	*M*	*SD*
Youth responses (RSQ proportion scores)		
Involuntary engagement	0.27	0.04
Involuntary disengagement	0.20	0.03
Secondary control coping	0.19	0.05
Primary control coping	0.18	0.02
Disengagement coping	0.16	0.02
Perceived effectiveness of youth coping strategies		
Avoidance/leaving situations	2.99	0.80
Problem-solving/environmental change	2.88	0.80
Distraction/attention shift	2.13	0.97
Emotional expression/support seeking	2.00	1.07
Advocacy/communication	1.96	1.06
Emotion regulation/calming	1.91	1.07
Thinking strategies	1.77	1.28
Rumination/getting stuck on misophonia-related thoughts	1.18	1.20
Parent responses (FAS frequency scores)		
Modifying own behavior	3.24	1.06
Assisting in avoidance of potential triggers	3.15	0.99
Providing reassurance	2.76	1.46
Modifying family routines	2.69	1.41
Providing needed items (e.g., headphones)	2.41	1.35
Avoiding doing things, going places, or being with people	2.36	1.31
Taking over child’s responsibilities	1.69	1.37
Modifying own leisure activities	1.61	1.25
Modifying work schedule	1.38	1.17
Perceived effectiveness of parent responses		
Modifying own behavior	2.48	1.06
Assisting in avoidance of potential triggers	2.44	1.08
Modifying family routines	2.41	1.02
Providing needed items (e.g., headphones)	2.40	0.92
Avoiding doing things, going places, or being with people	2.24	1.01
Taking over child’s responsibilities	2.15	1.01
Providing reassurance	2.08	1.26
Modifying own leisure activities	2.00	1.10
Modifying work schedule	1.95	1.04

Note. RSQ = Responses to Stress Questionnaire; FAS = Family Accommodation Scale. RSQ domain values are reported as proportion scores. FAS frequency scores range from 0 to 4, with higher scores indicating more frequent parent responses. Percieved effectiveness of youth coping strategies and parent responses were rated from 0 (*not at all helpful*) to 4 (*extremely helpful*), with higher scores indicating greater perceived helpfulness.

**Table 3:** Descriptive statistics and correlations among study variables.

Variable	*M*	*SD*	1	2	3	4	5	6	7	8	9	10	11	12
1. A-MISO-S	11.25	3.44	—											
2. DMQ Symptom Severity	50.12	14.64	0.81 ***	—										
3. DVMSQ	44.86	13.55	0.80 ***	0.76 ***	—									
4. MAQ	32.12	12.44	0.78 ***	0.72 ***	0.64 ***	—								
5. Primary Control Coping	0.18	0.02	−0.37 ***	−0.38 ***	−0.34 **	−0.50 ***	—							
6. Secondary Control Coping	0.19	0.05	−0.53 ***	−0.49 ***	−0.59 ***	−0.31 **	0.10	—						
7. Disengagement Coping	0.16	0.02	−0.21 *	−0.09	−0.21	0.01	−0.30 **	0.22 *	—					
8. Involuntary Engagement	0.27	0.04	0.54 ***	0.49 ***	0.57 ***	0.29 **	−0.22 *	−0.83 ***	−0.43 ***	—				
9. Involuntary Disengagement	0.20	0.03	0.56 ***	0.50 ***	0.60 ***	0.49 ***	−0.48 ***	−0.74 ***	−0.17	0.49 ***	—			
10. Parent Responses	21.31	7.21	0.55 ***	0.45 ***	0.47 ***	0.65 ***	−0.27 *	−0.14	−0.01	0.17	0.22 *	—		
11. Parenting Stress	40.78	11.07	0.47 ***	0.36 ***	0.45 ***	0.32 **	−0.36 ***	−0.37 ***	−0.11	0.42 ***	0.42 ***	0.17	—	
12. Family Functioning	1.70	0.48	0.04	0.03	0.06	0.11	−0.14	−0.18	0.01	0.16	0.22 *	−0.12	0.55 ***	—

Note. * *p* < 0.05. ** *p* < 0.01. *** *p* < 0.001. A-MISO-S = Amsterdam Misophonia Scale; DMQ = Duke Misophonia Questionnaire; DVMSQ = Duke-Vanderbilt Misophonia Questionnaire; MAQ = Misophonia Assessment Questionnaire.

## Data Availability

The data presented in this study are available upon reasonable request from the corresponding author.

## Supplementary Materials

The following supporting information can be downloaded at: https://www.mdpi.com/article/10.3390/jcm15207986/s1, File S1: Responses to Stress Questionnaire (RSQ) Adapted for Misophonia.
