# Supplementary material for: Characterizing How Families Cope with Pediatric Misophonia: Youth Coping, Parent Responses, and Associations with Symptom Severity
Source: J Clin Med. 2026 Oct 15;15(20):7986. doi: 10.3390/jcm15207986 (PMC13705739; doi:10.3390/jcm15207986)
Supplement: Supplementary file 1 [file jcm-15-07986-s001.zip › jcm-4572210-File S1.pdf]

ID: ______________
Date: _____________

## RESPONSES TO STRESS – [Parent-Report on Child] (PR-C)

This is a list of things about misophonia that children and teenagers sometimes find stressful or a problem to deal with. Please circle the number indicating how stressful you think the following things have been for your child in the past 6 months, as far as you know

| | Not at All | A Little | Somewhat | Very |
|---|---|---|---|---|
| a. Being upset or distressed by certain sounds (e.g., chewing, tapping, sniffing) | 1 | 2 | 3 | 4 |
| b. Avoiding meals with family or others because of sound triggers. | 1 | 2 | 3 | 4 |
| c. Arguments or conflict with family members because of sound triggers. | 1 | 2 | 3 | 4 |
| d. Your child feeling guilty for asking others to stop or change what they are doing. | 1 | 2 | 3 | 4 |
| e. Your child feeling misunderstood or not believed about misophonia. | 1 | 2 | 3 | 4 |
| f. Your child feeling embarrassed or ashamed about reactions to sounds | 1 | 2 | 3 | 4 |
| g. Teasing, bullying, or being left out because of misophonia | 1 | 2 | 3 | 4 |
| h. Difficulty concentrating at school or during homework because of sound triggers. | 1 | 2 | 3 | 4 |
| i. Not being able to participate in activities because of sound triggers. | 1 | 2 | 3 | 4 |
| j. Feeling constantly "on guard," worrying about when the next sound trigger will happen. | 1 | 2 | 3 | 4 |
| k. Feeling different from other kids because of misophonia | 1 | 2 | 3 | 4 |
| l. Worrying about how misophonia may affect their future. | 1 | 2 | 3 | 4 |
| m. Other: ____________________________________ | | | | |

**Circle the number that shows how much control he/she generally thinks he/she has over these problems.**

| 1 | 2 | 3 | 4 |
|---|---|---|---|
| None | A little | Some | A lot |

Below is a list of things that children and teenagers sometimes do, think, or feel when they are dealing with misophonia. Everyone deals with problems in their own way – some people do a lot of the things on this list or have a bunch of feelings, other people just do or think a few of these things.

**Think of all the stressful parts of misophonia that have been stressful for your child lately that you checked off above.** For each item below, circle **one** number from 1 (not at all) to 4 (a lot) that shows **how much** you think he/she does or feels these things when he/she has the problems with misophonia like the ones you indicated above. Please let us know about everything he/she does, thinks, and feels, even if you don't think it helps make things better.

***These problems may include both the everyday challenges of misophonia and what happens in the moment when a sound trigger occurs.***

| **WHEN DEALING WITH THE STRESS OF MISOPHONIA:** | **Not at all** | **A little** | **Some** | **A lot** |
|---|---|---|---|---|
| 1. He/she **tries** not to feel anything. | 1 | 2 | 3 | 4 |
| 2. When dealing with the stress of misophonia, he/she feels sick to his/her stomach or gets headaches. | 1 | 2 | 3 | 4 |
| 3. He/she tries to think of different ways to change or fix the situation. **Write one plan he/she thought of**:_______________________________________ | 1 | 2 | 3 | 4 |

(**How much does he/she do this?**)



Copyright © 2000 Bruce Compas. All rights reserved.

| WHEN DEALING WITH THE STRESS OF MISOPHONIA: | Not at all | A little | Some | A lot |
|---|---|---|---|---|
| | | | | |
| 4. When faced with the stress of misophonia, he/she doesn't feel anything at all, it's like he/she has no feelings. | 1 | 2 | 3 | 4 |
| 5. He/she wishes that he/she were stronger and less sensitive so that things would be different. | 1 | 2 | 3 | 4 |
| 6. He/she **keeps remembering** misophonia experiences (such as being triggered) or **can't stop thinking about** what might happen. | 1 | 2 | 3 | 4 |
| 7. He/she lets someone or something know how he/she feels, or lets someone know that a sound is bothering them. (*remember to circle a number.)* → **Check all he/she talked to:** ☐ Parent ☐ Friend ☐ Brother/Sister ☐ Pet ☐ Clergy Member ☐ Teacher ☐ God ☐ Stuffed Animal ☐ Other Family Member ☐ None of these | 1 | 2 | 3 | 4 |
| 8. He/she decides he/she is okay the way he/she is, even though he/she's not perfect. | 1 | 2 | 3 | 4 |
| 9. When he/she is around other people he/she acts like misophonia never happened. | 1 | 2 | 3 | 4 |
| 10. He/she just **has** to get away from everything when he/she is dealing with the stress of misophonia. | 1 | 2 | 3 | 4 |
| 11. He/she deals with the stress of misophonia by wishing it would just go away, that everything would work itself out. | 1 | 2 | 3 | 4 |
| 12. He/she gets really jumpy when he/she is dealing with the stress of misophonia. | 1 | 2 | 3 | 4 |
| 13. He/she realizes that he/she just has to live with things the way they are. | 1 | 2 | 3 | 4 |
| 14. When he/she is dealing with the stress of misophonia, he/she just **can't** be near anything that reminds him/her of what is happening. | 1 | 2 | 3 | 4 |
| 15. He/she **tries** not to think about it, to forget all about it. | 1 | 2 | 3 | 4 |
| 16. When he/she is dealing with the stress of misophonia, he/she really doesn't know what he/she feels. | 1 | 2 | 3 | 4 |
| 17. He/she asks other people or things for help or for ideas about how to make things better. (*remember to circle a number.*) → **Check all he/she talked to:** ☐ Parent ☐ Friend ☐ Brother/Sister ☐ Pet ☐ Clergy Member ☐ Teacher ☐ God ☐ Stuffed Animal ☐ Other Family Member ☐ None of these | 1 | 2 | 3 | 4 |
| 18. When he/she is trying to sleep, he/she **can't stop** thinking about misophonia or he/she has bad dreams about misophonia. | 1 | 2 | 3 | 4 |
| 19. He/she tells himself/herself that he/she can get through this, or that he/she will be okay. | 1 | 2 | 3 | 4 |
| 20. He/she lets his/her feelings out. (*remember to circle a number.*) → **He/she does this by: (Check all that he/she did.)** ☐ Writing in his/her journal/diary ☐ Drawing/painting ☐ Complaining to let off steam ☐ Being sarcastic/making fun ☐ Listening to music ☐ Punching a pillow ☐ Exercising ☐ Yelling ☐ Crying ☐ None of these | 1 | 2 | 3 | 4 |
| 21. He/she gets help from other people or things when he/she is trying to figure out how to deal with his/her feelings. (*remember to circle a number.*) → **Check all that he/she went to:** | 1 | 2 | 3 | 4 |

How much does he/she do this? (column heading)



Copyright © 2000 Bruce Compas. All rights reserved.

**Your half done. Before you keep working, look back at the first page so you remember the aspects of having misophonia that have been stressful for your child lately. Remember to answer the questions below thinking about these things.**

| WHEN DEALING WITH THE STRESS OF MISOPHONIA: | Not at all | A little | Some | A lot |
|---|---|---|---|---|
| | **How much does he/she do this?** | | | |
| ☐ Parent ☐ Friend ☐ Brother/Sister ☐ Pet ☐ Clergy Member<br>☐ Teacher ☐ God ☐ Stuffed Animal ☐ Other Family Member ☐ None of these | | | | |
| 22. **He/she just can't** get himself/herself to face misophonia stress or triggers | 1 | 2 | 3 | 4 |
| 23. He/she wishes that someone would just come and take away the stressful aspects of misophonia | 1 | 2 | 3 | 4 |
| 24. He/she does something to try to fix the stressful parts of misophonia (e.g., puts on headphones, changes seats or asks others to stop a sound).<br>**Check all that he/she does:**<br>☐ Put on headphones/ear protection<br>☐ Change seats or move to a different location ☐ Mimic or repeat the sound<br>☐ Ask others to stop or change the sound ☐ None of these<br>☐ Increase background noise (e.g., TV, white noise) ☐ Other: ____________ | 1 | 2 | 3 | 4 |
| 25. Thoughts about misophonia just pop into his/her head. | 1 | 2 | 3 | 4 |
| 26. When he/she is dealing with misophonia, he/she feels it in his/her body. (*remember to circle a number.)* →<br>**Check all that happen:**<br>☐ His/her heart races ☐ His/her breathing speeds up ☐ None of these<br>☐ He/she feels hot or sweaty ☐ His/her muscles get tight | 1 | 2 | 3 | 4 |
| 27. He/she **tries** to stay away from people and situations that might trigger or remind him/her of misophonia (e.g., avoids mealtimes, leaves the room, or skips activities where triggers might happen). | 1 | 2 | 3 | 4 |
| 28. He/she doesn't feel like himself/herself when he/she is dealing with misophonia, it's like he/she is far away from everything. | 1 | 2 | 3 | 4 |
| 29. He/she just takes things as they are; he/she goes with the flow. | 1 | 2 | 3 | 4 |
| 30. He/she thinks about happy things to take his/her mind off misophonia or how he/she is **feeling**. | 1 | 2 | 3 | 4 |
| 31. When something stressful happens related to misophonia, he/she **can't stop** thinking about how he/she is feeling. | 1 | 2 | 3 | 4 |
| 32. He/she gets sympathy, understanding, or support from someone. (*remember to circle a number.)* →<br>**Check all he/she went to:**<br>☐ Parent ☐ Friend ☐ Brother/Sister ☐ Pet ☐ Clergy Member<br>☐ Teacher ☐ God ☐ Stuffed Animal ☐ Other Family Member ☐ None of these | 1 | 2 | 3 | 4 |
| 33. When something stressful happens related to misophonia, he/she **can't** always control what he/she does. (*remember to circle a number.)* →<br>**Check all that happen:**<br>☐ He/she can't stop eating ☐ He/she can't stop talking<br>☐ He/she does dangerous things ☐ He/she has to keep fixing/checking things<br>☐ None of these | 1 | 2 | 3 | 4 |
| 34. He/she tells himself/herself that things could be worse. | 1 | 2 | 3 | 4 |
| 35. His/her mind just goes blank when something stressful happens related to misophonia, he/she can't think at all. | 1 | 2 | 3 | 4 |
| 36. He/she tells himself/herself that it doesn't matter, that it isn't a big deal. | 1 | 2 | 3 | 4 |



Copyright © 2000 Bruce Compas. All rights reserved.

| WHEN DEALING WITH THE STRESS OF MISOPHONIA: | How much does he/she do this? Not at all | A little | Some | A lot |
|---|---|---|---|---|
| 37. When he/she is faced with the stressful parts of misophonia, right away he/she feels really: (*remember to circle a number.)* → **Check all that he/she feels:** ☐ Angry ☐ Sad ☐ None of these ☐ Worried/anxious ☐ Scared ☐ Overwhelmed ☐ Irritated/annoyed | 1 | 2 | 3 | 4 |
| 38. It's really hard for him/her to concentrate or pay attention when something stressful happens related to misophonia. | 1 | 2 | 3 | 4 |
| 39. He/she thinks about the things he/she is learning from misophonia, or something good that will come from it. | 1 | 2 | 3 | 4 |
| 40. After something stressful happens related to misophonia, he/she **can't stop** thinking about what he/she did or said. | 1 | 2 | 3 | 4 |
| 41. When stressful parts of misophonia happen, he/she says to himself/herself, "This misophonia trigger isn't real." | 1 | 2 | 3 | 4 |
| 42. When he/she is dealing with the stressful parts of misophonia, he/she ends up just lying around or sleeping a lot. | 1 | 2 | 3 | 4 |
| 43. He/she keeps his/her mind off stressful parts of misophonia by: (*remember to circle a number.)* → **Check all that he/she does:** ☐ Exercising ☐ Seeing friends ☐ Watching TV ☐ Playing video games ☐ Doing a hobby ☐ Listening to music ☐ None of these | 1 | 2 | 3 | 4 |
| 44. When something stressful happens related to misophonia, he/she gets upset by things that don't usually bother him/her. | 1 | 2 | 3 | 4 |
| 45. He/she does something to calm himself/herself down when he/she is dealing with the stress of misophonia. (*remember to circle a number.)* → **Check all that he/she does:** ☐ Take deep breaths ☐ Pray ☐ Walk ☐ Listen to music ☐ Take a break ☐ Meditate ☐ Uses white noise ☐ Use sensory soothing tools (e.g., fidget, weighted item) ☐ None of these | 1 | 2 | 3 | 4 |
| 46. He/she just freezes when he/she is dealing with stressful parts of misophonia, he/she **can't** do anything. | 1 | 2 | 3 | 4 |
| 47. When stressful things happen related to misophonia, he/she sometimes acts without thinking. | 1 | 2 | 3 | 4 |
| 48. He/she keeps his/her feelings under control when he/she has to, then lets them out when they won't make things worse. | 1 | 2 | 3 | 4 |
| 49. When something stressful happens related to misophonia, he/she can't seem to get around to doing things he/she is supposed to do. | 1 | 2 | 3 | 4 |
| 50. He/she tells himself/herself that everything will be all right. | 1 | 2 | 3 | 4 |
| 51. When something stressful happens related to misophonia, he/she **can't stop** thinking about **why** this is happening. | 1 | 2 | 3 | 4 |
| 52. He/she thinks of ways to laugh about it so that it won't seem so bad. | 1 | 2 | 3 | 4 |
| 53. His/her thoughts start racing when he/she is faced with the stressful parts of misophonia. | 1 | 2 | 3 | 4 |
| 54. He/she imagines something really fun or exciting happening in his/her life. | 1 | 2 | 3 | 4 |



Copyright © 2000 Bruce Compas. All rights reserved.

| **WHEN DEALING WITH THE STRESS OF MISOPHONIA:** | **How much does he/she do this?** Not at all | A little | Some | A lot |
|---|---|---|---|---|
| 55. When something stressful happens related to misophonia, he/she can get so upset that he/she can't remember what happened or what he/she did. | 1 | 2 | 3 | 4 |
| 56. He/she tries to believe that the misophonia trigger never happened. | 1 | 2 | 3 | 4 |
| 57. When he/she is dealing with the stress of misophonia, sometimes he/she **can't** control what he/she does or says. | 1 | 2 | 3 | 4 |



Copyright © 2000 Bruce Compas. All rights reserved.
